# Epigallocatechin Gallate Nanodelivery Systems for Cancer Therapy

**DOI:** 10.3390/nu8050307

**Published:** 2016-05-20

**Authors:** Andreia Granja, Marina Pinheiro, Salette Reis

**Affiliations:** UCIBIO/REQUIMTE, Department of Chemical Sciences, Faculty of Pharmacy, University of Porto, Rua de Jorge Viterbo Ferreira, 228, 4050-313 Porto, Portugal; bio11041@fe.up.pt (A.G.); shreis@ff.up.pt (S.R.)

**Keywords:** green tea, EGCG, cancer, nanotechnology, nanochemoprevention, anti-cancer therapy

## Abstract

Cancer is one of the leading causes of morbidity and mortality all over the world. Conventional treatments, such as chemotherapy, are generally expensive, highly toxic and lack efficiency. Cancer chemoprevention using phytochemicals is emerging as a promising approach for the treatment of early carcinogenic processes. (−)-Epigallocatechin-3-gallate (EGCG) is the major bioactive constituent in green tea with numerous health benefits including anti-cancer activity, which has been intensively studied. Besides its potential for chemoprevention, EGCG has also been shown to synergize with common anti-cancer agents, which makes it a suitable adjuvant in chemotherapy. However, limitations in terms of stability and bioavailability have hampered its application in clinical settings. Nanotechnology may have an important role in improving the pharmacokinetic and pharmacodynamics of EGCG. Indeed, several studies have already reported the use of nanoparticles as delivery vehicles of EGCG for cancer therapy. The aim of this article is to discuss the EGCG molecule and its associated health benefits, particularly its anti-cancer activity and provide an overview of the studies that have employed nanotechnology strategies to enhance EGCG’s properties and potentiate its anti-tumoral activity.

## 1. Introduction

Cancer is a disease characterized by an excessive and uncontrolled growth of cells that can metastasize to several organs and eventually cause death of the host [1]. This disease is one of the leading causes of morbidity and mortality all over the world [2]. In 2012, approximately 14.1 million new cases were diagnosed and 8.2 million cancer-related deaths occurred worldwide [3]. By 2025, 19.3 million new cases are expected to emerge each year [4]. The costs associated with cancer are also a major matter of concern. In 2013, the total healthcare expenditure associated with cancer in the US was $74.8 billion [1]. Conventional treatments for the disease include surgery, hormone therapy, radiation and chemotherapy [1]. Chemotherapy is the main treatment for most cancers in advanced stage [5]. This therapeutic has, however, several limitations such as high costs, lack of efficiency and elevated toxicity, causing various side effects, including anemia, exhaustion, nausea and hair loss, which greatly impacts quality of life [5,6,7]. Therefore, it is essential to explore and develop novel strategies to minimize the undesirable effects of chemotherapy and increase its anti-cancer efficacy [5].

The use of natural compounds, such as phytochemicals has emerged as a potential strategy for cancer management. These compounds are of great interest due to their high spectrum of biological activity, low cost and minimal side effects [8,9]. One popular phytochemical with great potential is found in green tea, which is a healthy beverage consumed worldwide and produced from the leaves of *Camellia sinensis* [8,10]. (−)-Epigallocatechin-3-gallate (EGCG) is the most abundant and the most biologically active catechin in green tea and its role in cancer treatment has been intensively studied [11]. EGCG chemopreventive and chemotherapeutic activity has been demonstrated in several *in vitro* and *in vivo* animal studies [12,13,14,15,16]. The results have also been corroborated by various epidemiological and preclinical studies, which demonstrated a correlation between green tea regular consumption and cancer prevention and the inhibition of tumor progression [17,18,19,20,21]. In addition, EGCG offers several advantages over conventional therapies since it is widely available and inexpensive to isolate from green tea, it can be administered orally and it has an acceptable safety profile [22]. Despite its enormous potential as an anti-cancer agent, EGCG has a short half-life, low stability and low bioavailability, greatly limiting its use in clinical settings [8,23]. In a study developed by Nakagawa *et al.* [24] EGCG levels detected in plasma corresponded to only 0.2%–2% of the ingested amount. In addition, the effective anti-tumoral concentration of EGCG *in vitro* is generally an order of magnitude higher than the levels measured *in vivo*, which restricts its effectiveness [8]. Moreover, EGCG lacks target specificity [23]. Therefore, a strategy that increases EGCG stability and bioavailability and simultaneously targets cancer cells is necessary. Recently, the concept of nanochemoprevention was introduced [25]. This strategy consists of the use of nanotechnology to improve the pharmacokinetic and pharmacodynamic of chemopreventive agents in order to prevent, slow-down or revert cancer [25]. EGCG encapsulation into a specific nanocarrier can increase its solubility and bioavailability, protect it from premature degradation, prolong its circulation time and induce higher levels of target specificity due to the possibility of nanoparticle (NP) surface functionalization [25]. Several studies have already implemented this strategy encapsulating EGCG into different types of nanoparticles for cancer treatment [25].

The aim of this article is to provide a critical review of the EGCG molecule and its associated health benefits with a special focus on its anti-cancer activity. In addition, an overview of the applications that used nanotechnology strategies to deliver EGCG to cancer cells will also be given.

## 2. EGCG

### 2.1. Source and Chemical Structure

**Figure 1:**
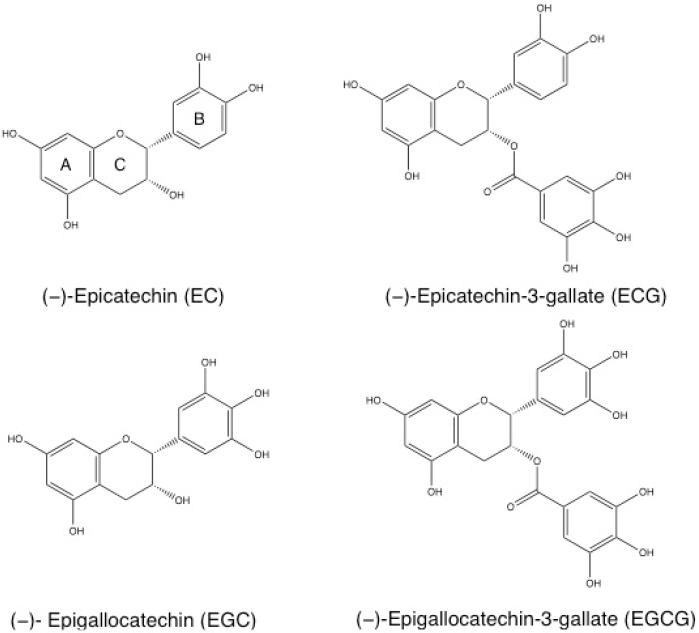
Chemical structure of (−)-Epicatechin (EC), (−)-Epicatechin-3-gallate (ECG), (−)-Epigallocatechin (EGC) and (−)-Epigallocatechin-3-gallate (EGCG).

Green tea is composed of different chemical compounds, such as amino acids, vitamins, inorganic elements, carbohydrates, lipids, caffeine and tea polyphenols [26]. Polyphenols constitute about 30% of the dry weight of green tea leaves and are the main compound responsible for its health promoting effects [27]. Catechins form the major group of polyphenols found in green tea and comprise different molecules such as (−)-Epicatechin (EC), (−)-Epicatechin-3-gallate (ECG), (−)-Epigallocatechin (EGC), and (−)-Epigallocatechin-3-gallate (EGCG) [28]. The chemical structures of catechins are represented in Figure 1.

These molecules are composed of a polyphenolic structure that allows electron delocalization, enabling the quenching of free radicals [29]. Catechins are characterized by a dihydroxyl or trihydroxyl substitution on the B ring, a meta-5, 7-dihydroxyl substitutions on the A ring and, in the case of the galloylated catechins ECG and EGCG, the trihydroxyl substitutions on the D ring [29]. EGCG is the major catechin and the most biologically active compound, accounting for 50%–80% of the total catechins in green tea [5,12]. This molecule has a trihydroxyl substitution on the B ring and a gallate moiety esterified at carbon 3 on the C ring [28]. These structural characteristics contribute to its increased anti-oxidant and iron-chelating activities [28]. Tea catechins, particularly EGCG, have several pharmacological and biological properties, such as anti-oxidant, free radical scavenging [30,31], anti-bacterial [32,33], anti-viral [34,35,36], anti-diabetic [37,38,39,40], cardioprotective, anti-atherosclerotic, anti-inflammatory [41,42,43,44,45,46], anti-obesity [47], neuroprotective [48,49,50] and anti-carcinogenic effects [12,13,14,15,16,17,18,19,20,21]. The latter, in particular, has been intensively studied [12,13,14,15,16,17,18,19,20,21].

### 2.2. Anti-Cancer Activity

EGCG has been shown to play a significant role as an anti-cancer agent. Cancer is a disease characterized by an abnormal growth of cells, which generates excessive cell proliferation over cell death [51]. This imbalance culminates in the formation of a group of cells that can invade tissues and metastasize to distant regions, causing morbidity and, eventually, death of the host [51]. Cancer is associated with multiple changes in gene expression, which affect the normal mechanisms of cell division and differentiation [51]. The factors that trigger these alterations are not clearly defined in most cases, however, it is established that both external (such as an unhealthy diet, chemicals, tobacco and radiation) and internal (such as inherited genetic mutations and immune conditions) factors may have an impact in the onset of the disease [51]. EGCG’s anti-tumoral effects have been demonstrated both in cell culture and animal experiments and in epidemiological and clinical studies [12,13,14,15,16,17,18,19,20,21].

**Figure 2:**
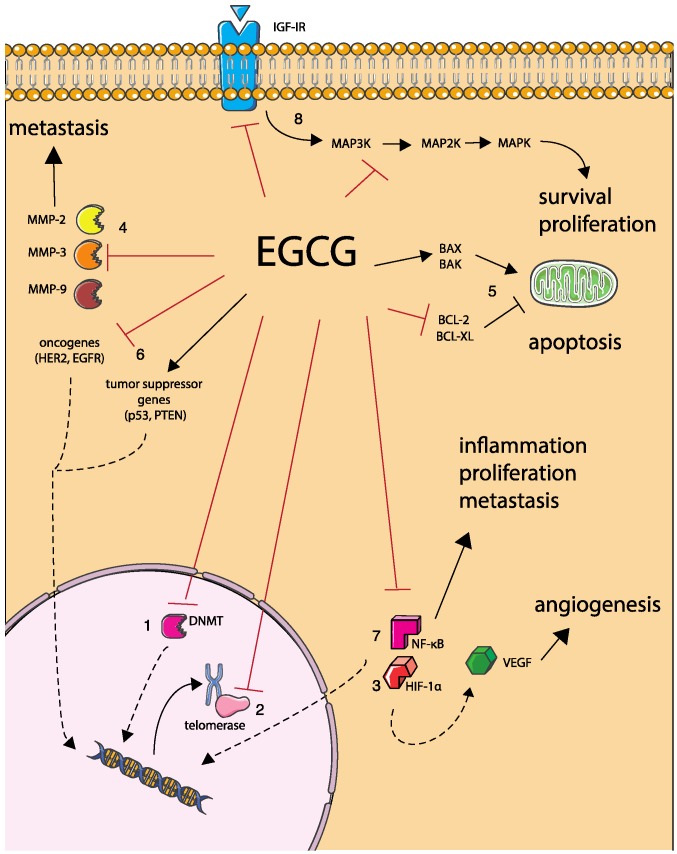
Cancer-related cell mechanisms modulated by EGCG: (**1**) Inhibition of DNA hypermethylation by direct blocking of DNA methyltransferase (DNMT); (**2**) Repression of telomerase activity; (**3**) Inhibition of angiogenesis by repression of transcription factors Hypoxia-inducible factor 1-α (HIF-1α) and Nuclear factor kappa B (NF-κB); (**4**) Blocking of cell metastasis by inhibition of Matrix metalloproteinases (MMPs) -2, -9 and -3; (**5**) Promotion of cancer cell apoptosis by induction of pro-apoptotic proteins BCL-2-associated X protein (BAX) and BCL-2 homologous antagonist killer (BAK) and repression of anti-apoptotic proteins B-cell lymphoma 2 (BCL-2) and B cell lymphoma-extra large (BCL-XL); (**6**) Induction of tumor suppressor genes *p53* and *Phosphatase and tensin homolog* (*PTEN)* and inhibition of oncogenes *Human epidermal growth factor receptor 2* (*HER2*) and *Epidermal growth factor receptor* (*EGFR*); (**7**) Inhibition of NF-κB and subsequent events of cell inflammation, proliferation, metastasis and angiogenesis; and (**8**) Anti-proliferative activity by inhibition of Mitogen-activated protein kinases (MAPK) pathway and Insulin-like growth factor I receptor (IGFIR).

EGCG is involved in numerous signaling pathways and biological mechanisms related with cancer development and progression (Figure 2), discussed in more detail below.

#### 2.2.1. DNA Hypermethylation

DNA methylation is a biochemical modification that consists of the addition of a methyl group to a cytosine within a CpG site, a process that is performed by the enzyme DNA methyltransferase (DNMT) [52]. Hypermethylation usually inhibits the binding of the transcription factors to the promoter region, which induces gene silencing [53]. This process occurs frequently during cancer development with inhibition of cell cycle regulator, receptor and apoptotic genes [54]. It has been demonstrated that EGCG has the ability to directly block DNMTs, and consequently, restore the expression of these genes, which may have an impact on cancer progression [55].

#### 2.2.2. Telomerase Activity

Telomeres are regions localized at the end of eukaryotic chromosomes responsible for DNA protection and genomic stability [56]. Telomerase is a reverse transcriptase responsible for telomere preservation [56]. These enzymes were found to be upregulated in various types of tumors [57]. Different studies demonstrated the capacity of EGCG to inhibit telomerase activity in different cancer cell lines including lung carcinoma [58], cervical cancer [59], leukemia and adenocarcinoma cells [60], thus emphasizing its potential to block the development and progression of these tumors.

#### 2.2.3. Angiogenesis

Tumor angiogenesis is one of the hallmarks of cancer with a huge impact on tumor progression [61]. It consists of the recruitment of blood vessels to the tumor site, to assure oxygen and nutrient supply [62]. Angiogenesis is stimulated by several different factors, including Vascular Endothelial Growth Factor (VEGF) [63]. Various studies described that EGCG can significantly inhibit VEGF expression through repression of transcription factors Hypoxia-inducible factor 1-α (HIF-1α) and Nuclear factor kappa B (NF-κB), thus suppressing angiogenesis [64,65,66]. *In vivo* studies using nude mice also corroborated this capacity, showing an inhibition of vascularity and tumor growth and proliferation after treatment with EGCG [65,67].

#### 2.2.4. Metastasis

Another cancer hallmark is cell metastasis, which is an extension of cell invasion [62]. After invasion, cancer cells can pass through the extracellular matrix and enter into the bloodstream, being able to disseminate and create a new niche in another location, forming a metastatic focus [61]. To metastasize, tumor cells have to degrade the basement membrane and the stroma, which is possible through the secretion of specific proteases called Matrix metalloproteinases (MMPs) [61]. Inhibition of these MMPs has been revealed to inhibit metastasis and tumor growth in mouse xenograft models [61]. EGCG has demonstrated ability to prevent cancer cell metastasis, due to inhibition of matrix MMPs -2, -3 and -9, which play an important role in metastasis, via direct binding and gene expression repression [68,69,70,71].

#### 2.2.5. Cancer Cell Apoptosis

Apoptosis is the process of programmed cell death that often culminates in the activation of cysteine-aspartic proteases (caspases), which are responsible for the cleavage of intra-cellular proteins triggering sequential events that will culminate into induction of cell death [72]. Two main pathways can induce this event: extrinsic and intrinsic pathway [72]. In the extrinsic pathway, apoptosis is triggered by the binding of death ligands to death receptors, which induces intra-cellular signaling mechanisms that activate caspases [72]. In the intrinsic pathway, activation of pro-apoptotic proteins BCL-2-associated X protein (BAX) and BCL-2 homologous antagonist killer (BAK) promotes the release of proteins from the mitochondria leading to the formation of the apoptosome and culminating in the activation of caspases [72]. The regulation of this pathway is done by apoptosis inhibitors, such as B-cell lymphoma 2 (BCL-2) and B cell lymphoma-extra large (BCL-XL), which antagonize with BAX and BAK [72]. The apoptotic pathways described above are often downregulated in cancer [72,73]. As a consequence, apoptosis has been widely studied as a target for anti-cancer therapies [72]. Different studies have demonstrated that EGCG can inhibit the expression of the anti-apoptotic proteins BCL-2 and BCL-XL and induce the expression of apoptotic proteins BAX and BAK, with subsequent activation of caspases in several types of cancers [73,74,75,76]. In addition, EGCG has revealed ability to induce H_2_O_2_ production [77], block cell cycle progression [78] and inhibit NF-κB [79,80], events which will also induce apoptosis.

#### 2.2.6. Tumor Suppressor Genes and Oncogenes Expression

Tumor suppressor genes are genes that reduce the probability of a normal cell to become a tumor cell [81]. These genes are usually associated with cell cycle arrest and apoptosis induction triggered by DNA damage [81]. Mutations in tumor suppressor genes severely increase the probability of cancer development [81]. In fact, their inactivation has been observed in several types of tumors [81]. EGCG has revealed capacity to increase the expression of tumor suppressor gene *p53* [82,83] and *Phosphatase and tensin homolog* (*PTEN*) [84] and cyclin-dependent kinase inhibitors *p21* and *p27* [83,85] in different cancer cell lines, including breast, pancreas and prostate cancer. Oncogenes are mutated genes that have influence on the development of cancer [86]. There are several types of oncogenes, whose function is usually associated with cell proliferation, such as *Epidermal growth factor receptor (EGFR)* and *Human epidermal growth factor receptor 2 (HER2)*. These genes are frequently overexpressed in several types of cancers [87,88]. Some studies revealed that EGCG is able to inhibit the activation of HER2 and EGFR in different cancer cells lines, such as, lung, thyroid, breast cancer and squamous-cell carcinoma [89,90,91,92].

#### 2.2.7. NF-κB Activation and Nuclear Translocation

NF-κB is a family of transcription factors activated by numerous stimuli, amongst them free radicals, inflammatory signals, cytokines, carcinogens, UV-light and tumor promoters [93]. After activation, NF-κB migrates to the nucleus and induces the expression of genes responsible for the suppression of apoptosis, inflammation, proliferation and metastasis [93]. Different studies showed that EGCG can efficiently inhibit the activation and nuclear translocation of this transcription factor, preventing the subsequent events related to cancer progression in different types of tumor cell lines, including epidermoid carcinoma cells [94], bladder [95], breast, and head and neck [96] cancer cells.

#### 2.2.8. Anti-Proliferative Activity

EGCG revealed anti-proliferative ability on cancer cells by inhibiting mitogenic signal transduction pathways. Mitogen-activated protein kinases (MAPK) are protein kinases involved in the cytoplasmic phase of the signaling pathway initiated by the binding of growth factor to a transmembrane receptor [97]. These pathways are responsible for cell survival and proliferation and are highly related to cancer development [97]. EGCG has proven its ability to inhibit MAPK pathway in different cancer cell types, such as colon [98], endometrial [99] and leukemia [100]. In addition EGCG was shown to directly bind and inhibit Insulin-like growth factor I receptor (IGFIR) activity, which is one of the receptors than can lead to activation of the MAPK pathway and plays an important role in cell proliferation [101,102].

#### 2.2.9. Protein Binding

The anticancer effects of EGCG may be explained in part due to its capacity to bind directly to several proteins involved in different cell mechanisms such as proliferation, apoptosis and metastasis. Suzuki *et al.* showed that EGCG can bind to plasma protein fibrinogen and cell adhesive proteins fibronectin and laminin [103,104]. These interactions may be related to the capacity of EGCG to inhibit metastasis [105]. EGCG has also been shown to directly bind to Fas, triggering Fas-mediated apoptosis [106]. This may be one of the main mechanisms by which EGCG induces apoptosis in cancer cells [106]. Tachibana *et al.* identified 67-kDa laminin receptor as a mediator of EGCG anticancer effects (67LR) [107]. Ermakova *et al.* [108] demonstrated that EGCG binds to vimentin, a protein responsible for mitosis, locomotion and structural integrity, and inhibits its phosphorylation, decreasing cell proliferation. The same authors found other relevant proteins inhibited by EGCG via direct binding such as the chaperone protein glucose-regulated protein 78 (GRP78), whose anti-apoptotic effects are related to chemotherapeutic drug resistance [109], IGFIR, highly associated with cell proliferation and cancer development [102] and the tyrosine kinases Fyn [110] and ZAP-70 [111]. Other EGCG-binding proteins were also identified such as Ras-GTPase-activating protein SH3 domain-binding protein 1 (G3BP1) [112] and peptidyl prolyl cis/trans isomerase (Pin1) [113], both involved in oncogenic cell signaling pathways.

#### 2.2.10. *In Vivo* Experiments

Inhibition of tumorigenesis by EGCG was also demonstrated *in vivo* in mice models for different types of cancer, including breast [13], lung [14], intestine [15], skin [16] and prostate [114].

#### 2.2.11. Clinical Studies

Different clinical studies have corroborated the *in vitro* results. Patients with papilloma virus-infected cervical lesions were treated with 200 mg capsules of EGCG or green tea extracts and the treatment demonstrated effectiveness, with a 69% response rate [115]. Bettuzzi *et al.* demonstrated that daily administration of 600 mg of EGCG was effective in treating premalignant lesions in men with high-grade prostate intraepithelial neoplasia [18]. Consistent with this, McLarty *et al.* developed a phase II clinical trial in prostate carcinoma patients demonstrating a significant reduction in the levels of different cancer-related biomarkers in serum after oral administration of 800 mg of EGCG [116]. On the other hand, in a phase II study after administration of daily doses of EGCG to 42 androgen independent prostate cancer patients, only limited antineoplasic activity was detected [117].

#### 2.2.12. Epidemiological Data

Different epidemiological studies have addressed the effects of green tea and particularly EGCG, in prevention and treatment of cancer, further supporting the *in vitro* and *in vivo* results. A prospective cohort study with over 8000 individuals found that daily consumption of green tea delayed cancer onset [17]. Additionally, a follow up study with stages I and II breast cancer patients, determined lower recurrence rate and longer disease-free period after daily consumption of green tea [17]. Green tea daily consumption has also demonstrated a preventive effect against prostate cancer [19]. A prospective cohort study also revealed that green tea consumption is inversely associated with distal gastric cancer occurrence among women [20]. In this study, participants who consumed five or more cups per day had 49% less risk of having gastric tumors in the distal portion compared with the ones who drank less than 1 cup per day [20]. More recently, the protective role of green tea against stomach cancer was also demonstrated in a meta-analysis, where a reduction of 14% in the risk of stomach cancer with high green tea consumption was determined [21]. On the other hand, there are also many studies where weak or no association between cancer risk and green tea consumption was found as reported by Zhou *et al.* [118], Lin *et al.* [119] and Sasazuki *et al.* [120] For a more detailed review on this subject, see [121]. These differences in results may be explained in part by the low levels of EGCG present in the blood following green tea consumption, which may be insufficient to induce a chemopreventive effect [121].

The vast majority of these studies highlight the importance of EGCG in cancer and the pertinence of exploiting it in anti-tumoral therapy. Conventional treatments against cancer often consist of the administration of cytostatic drugs, which present several limitations. One of the most relevant is the lack of precision, which implies that only a small part of the drug reaches the tumor region, reducing the efficacy of the drug and causing systemic toxicity [122]. Another drawback is the fact that the drugs are also toxic to healthy cells, including bone marrow and gastrointestinal cells [122]. All these factors contribute to the well-known side effects associated with chemotherapy such as nausea, fatigue and hair loss [122]. EGCG can be used as an adjuvant in chemotherapy [123] lowering the doses of the cytostatic drugs used in chemotherapy and, consequently, the associated toxicity and side effects.

## 3. Nanotechnology and Nanochemoprevention

Nanotechnology is an interdisciplinary field that comprises the areas of biology, engineering, chemistry and medicine and relies on the use of nanosystems, which are man-made devices with at least one dimension in the range of 1–100 nanometers [124]. Nanotechnology is currently being studied and implemented in diagnosis and treatment of cancer, with the development of nanosensor devices and nanovectors [124]. Nanovectors include nanoparticles (NPs) for loading drugs or imaging agents and subsequent delivery and targeting to tumor cells [124]. A wide variety of different nanoparticles may be applied to develop anti-cancer drug delivery systems, including liposomes, magnetic NPs, polymeric NPs, among many others [124]. The potential of nanoparticles as anti-cancer drug delivery systems is enormous since they increase the absorption, solubility and bioavailability of the drug, protect it from premature degradation and extend its circulation time [23,124,125]. In addition, NPs can increase drug retention in tumor tissues, due to the enhanced permeability and retention effect (EPR), facilitate intra-cellular penetration, increase target specificity due to the possibility of surface functionalization and minimize drug toxic effects [23]. Furthermore, they enable oral administration of the drug, which is the preferred delivery route in terms of patient compliance and convenience [126].

**Figure 3:**
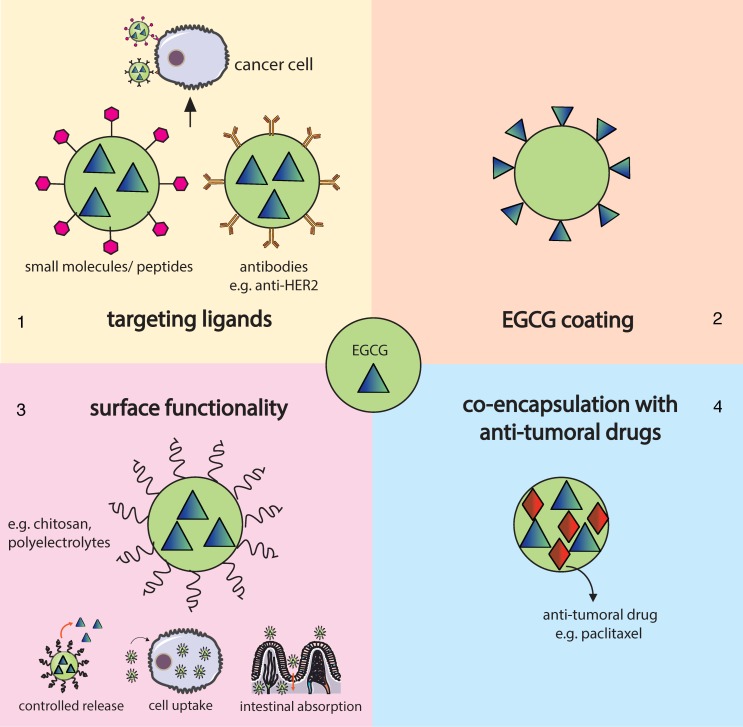
Summary of EGCG delivery approaches for cancer therapy reported in the literature: (**1**) incorporation of ligands (small molecules, peptides and antibodies) at the surface of the nanoparticle to target specific cancer cell receptors or antigens; (**2**) use of EGCG as a capping agent; (**3**) surface functionalization with specific polymers to enhance drug release properties, cell uptake and intestinal absorption; and (**4**) co-encapsulation with common cytostatic drugs such as paclitaxel.

Chemoprevention is a promising strategy that consists of the use of natural and synthetic compounds, such as EGCG, as a strategy for cancer prevention, slowdown or reversion [124]. Despite its potential, the efficiency of this approach is still limited due to toxicity and ineffective systemic delivery and bioavailability [25]. To overcome these limitations, Siddiqui *et al.* [25] introduced the concept of nanochemoprevention, which consists of the use of nanotechnology to improve the pharmacokinetic and pharmacodynamic of chemopreventive agents in order to manage cancer. In addition to chemopreventive applications, EGCG may also have a relevant role as an adjuvant in chemotherapy. Indeed, EGCG has already been shown to synergize with common anti-cancer agents such as doxorubicin, tamoxifen and paclitaxel in multiple cell lines [123]. Several studies reported in the literature have already applied nanotechnology strategies, using different types of nanoparticles as delivery vehicles of EGCG to target different types of cancer both *in vitro* and *in vivo*. These reports are discussed below in more detail grouped according to the type of nanoparticle used. The main strategies followed are schematically represented in Figure 3.

### 3.1. Gold Nanoparticles

Gold nanoparticles present unique physicochemical properties, such as small size, plasmon resonance, capacity to bind amine and thiol groups, high atomic number and biocompatibility [127]. Synthesis of these NPs usually involves the reduction of Au (III) derivatives, such as Chloroauric acid (HAuCl_4_) [127]. Generally, an aqueous solution of HAuCl_4_ is mixed with an aqueous solution of a reducing agent, which leads to the reduction of Au^3+^ and formation of gold nanoparticles [127]. Polyphenols may act as both reducing and capping agents of this process as reported by Nune *et al.* [128]. This approach avoids the use of an additional synthetic chemical reagent, which makes it a green chemistry process [128].

Due to their distinctive properties, gold NPs have been exploited in several biomedical applications as biosensors, contrast agents, drug delivery vehicles and anti-tumoral agents [125,129]. Gold NPs are suitable anti-cancer agents mainly due to their small size, which enables them to penetrate in the tissues and accumulate in the tumor site and their optical properties, which allow their use in photothermal anti-cancer therapies [125].

Several reports have described the effect of gold NPs in conjugation with EGCG for cancer treatment. The main results of these studies, including nanoparticle type, size, zeta potential, loading capacity (LC) encapsulation efficiency (EE) and *in vitro* and *in vivo* evaluation are summarized in Table 1.

Hsieh *et al.* [130] coated gold NPs with EGCG (EGCG-pNG) through an ultrasonication process and tested their effect in the treatment of bladder cancer both *in vitro* and *in vivo*. Their results showed that this strategy induced high levels of cytotoxicity in bladder cancer cells (MBT-2) without affecting the viability of normal cells (Vero cells). Treatment with EGCG-pNG was shown to induce apoptosis through triggering the intrinsic apoptotic pathway via the activation of caspases-3 and -7. *In vivo* tests confirmed these results. C3H/HeN mice subcutaneously implanted with MBT-2 cells revealed a significantly higher reduction in tumor volume after oral administration of EGCG-pNG in comparison with free EGCG. In addition, NPs were also administered via intra-tumoral and intra-peritoneal. These previous two administration routes were more effective than oral administration in suppressing tumor growth. In a more recent work, the same group [125] tested the efficiency of similar NPs against melanoma both *in vivo* and *in vitro*. *In vitro* results showed that gold NPs induced 4.91 times higher levels of apoptosis in B16F10 murine melanoma cells compared to non-encapsulated EGCG. Apoptosis was caused by activation of a mitochondrial-mediated pathway. This nanocarrier also demonstrated a high biocompatibility, inducing low damage to human red blood cells. *In vivo* results demonstrated that intra-tumoral injection of EGCG NPs induced a reduction in the tumor volume of a mouse melanoma model compared with the control treatment. This ability to inhibit tumor growth was 1.66 times higher when EGCG was encapsulated compared to free EGCG.

Sanna *et al.* [131] synthesized gold NPs using a similar process to the one described by Nune *et al.* [128]. EGCG-conjugated gold nanoparticles revealed high stability in simulated biological fluids and were able to retain EGCG’s anti-oxidant activity [131]. In addition, the nanoparticles were efficient in inducing apoptosis (through activation of caspase-3) in neuroblastoma SH-SY5Y-CFP-DEVD-YFP cells in a concentration dependent-manner after 72 h of exposure. The authors concluded that the efficiency of EGCG was maintained after adsorption to the surface of gold NPs. The same chemical process for the synthesis of gold NPs was replicated recently by Mukherjee *et al.* also with encouraging results [11]. EGCG-conjugated gold NPs revealed higher anti-oxidant activity, cellular internalization and cytotoxicity towards tumor cells than EGCG in a free form. At the same dose (20 μg/mL), EGCG NPs induced 30% more cell death in Ehrlich’s Ascites Carcinoma (EAC) cells than native EGCG. Apoptosis was induced due to an increase in lipid peroxidation and in the levels of ROS. A reduction in the levels of anti-oxidant enzymes, such as glutathione was observed as well as an inhibition of the nuclear translocation of the transcription factor NF-κB and subsequent activation of its downstream survival molecules. On the other hand, in normal primary mouse hepatocytes, EGCG NPs promoted an increase in the levels of anti-oxidant enzymes, protecting the cells against tumor-induced cellular damage. The results revealed that these NPs are able to induce tumor cell apoptosis and simultaneously protect hepatocytes against undesirable effects.

### 3.2. Polymeric Nanoparticles

Polymeric NPs present important characteristics, which make them suitable for biomedical applications, such as biocompatibility, biodegradability, with the possibility of controlling the rate of polymer degradation, mechanical strength, and high structure versatility [132,133].

Several polymers, natural or synthetic, can be employed to produce polymeric NPs, the most common include polycaprolactone (PCL), polylactic acid (PLA), poly (lactic-co-glycolic acid) (PLGA), chitosan and gelatin [134]. PLA and PLGA are approved and recognized as safe by the US Food and Drug Administration (FDA) for human applications and are metabolized in the organism into biodegradable biocompatible monomers (lactic and glycolic acid) [134]. Intravenous injection of PLGA and PLA usually leads to their rapid clearance by the immune system [25]. To increase their circulation time, NPs are frequently coated with PEG, also approved by the FDA, which stabilizes and avoids their recognition by the immune system [25]. Chitosan is a natural polymer characterized by its non-toxic, non-immunogenic and mucoadhesive properties in the gastrointestinal tract, which makes it suitable for oral routes of administration [126]. Gelatin is intensively used in food and medical products and it is also a non-toxic biodegradable polymer [134]. It is characterized by its mechanical, thermal and swelling properties, which are highly dependent on the degree of crosslinking [134]. Several groups have already encapsulated EGCG into different polymeric NPs for cancer therapy. The main findings from these studies are shown in Table 2.

Sanna *et al.* [135] designed EGCG-loaded PLGA-PEG NPs for treatment against prostate cancer. In this study, the function of the NPs was enhanced with a prostate-specific membrane antigen (PSMA) ligand (DCL). These NPs allowed a greater control of the rate of release of EGCG relative to that of free EGCG. Encapsulation and functionalization with DCL increased the cytotoxicity of the NPs towards LNCaP prostate cancer cell line, which were PSMA-positive. On the other hand, no significant inhibition of cell growth inhibition was detected in HUVECs (human umbilical vein endothelial cells). These results suggest that PLA-PEG-DCL EGCG-loaded NPs were able to efficiently kill PSMA-positive prostate cancer cells without influencing the viability of normal cells.

Alotaibi *et al.* [132] also prepared PLGA NPs for EGCG encapsulation. The DNA damage effect of these NPs was tested against lymphocytes of healthy and colorectal cancer patients pretreated with oxaliplatin of satraplatin. The obtained results suggest that encapsulated EGCG significantly intensified DNA damage levels in a dose-dependent way. In contrast, free EGCG promoted a reduction in DNA damage. The authors suggested that this catechin might alternate between an anti-oxidant (bulk form) and a pro-oxidant (encapsulated form) state.

Narayanan *et al.* [136] synthesized PLGA-casein NPs constituted by a core and a shell, where paclitaxel and EGCG, respectively, were entrapped. This organization enabled a sequential and controlled release of both drugs. Nanocarriers revealed a longer circulatory lifespan and increased biocompatibility both *in vitro* and *in vivo*. In a more recent study, the same authors tested the chemotherapeutic effect against breast cancer cells (MDA-MB-231 cells and patient-derived tumor cells) [137]. With that purpose some of the NPs were functionalized with antibodies specific for the cell surface receptors anti-EGFR and anti-HER2. The results showed an enhanced cellular uptake by MDA-MB-231 cells and a higher rate of apoptosis compared with individually encapsulated paclitaxel and EGCG. Both results were improved when NPs were functionalized with anti-EGFR. This therapy also showed an inhibitory effect in the protein levels of NF-κB, a signaling molecule activated by paclitaxel that may interfere with chemotherapy effectiveness, promoting angiogenesis, metastasis and drug resistance. Combination treatment functionalized with both EGFR and HER2 antibodies towards breast cancer samples from patients also showed significantly higher anti-tumoral activity.

Siddiqui *et al.* [25] reported the use of PLA-PEG NPs to encapsulate EGCG. The efficiency of NPs against human prostate cancer was determined both *in vitro* and *in vivo*. The *in vitro* results showed that EGCG NPs induced the same extent of cellular death in human prostate cancer PC3 cells as non-encapsulated EGCG with an over 10-fold dose advantage. These NPs promoted an increase in pro-apoptotic molecules, such as BAX and a decrease in anti-apoptotic molecules, such as BCL-2 confirming the ability that the NPs have to retain EGCG’s biological activity even at very low concentrations. Furthermore, EGCG-loaded NPs were also able to efficiently inhibit angiogenesis. This data was validated by *in vivo* results where it was observed that treatment with EGCG NPs induced a significant decrease in the tumor volume of athymic nude mice injected with androgen-responsive 22Rν1 cells with a 10-fold lower dose. More recently the same group [126], developed an EGCG nanocarrier specifically designed for oral administration using water-soluble chitosan. In this study, chitosan NPs revealed stability in an acidic environment, inducing a very slow release of EGCG in simulated gastric juice and a faster release in neutral pH (simulated intestinal fluid). The *in vivo* results determined in a prostate cancer xenograft model showed a significantly higher inhibition of tumor growth compared with both control and free EGCG-treated groups. This inhibition was found to be dose-dependent. Other relevant *in vivo* results include: inhibition of serum prostate cancer marker PSA; activation of DNA damage-related protein PARP; activation of mitochondrial pathway of apoptosis, with increase in the levels of pro-apototic protein BAX, decrease in the levels of anti-apoptotic protein BCL-2 and activation of caspases -3, -8 and -9, inhibition of cell proliferation markers (Ki-67 and PCNA) and angiogenesis markers (CD31 and VEGF). This oral nanoformulation with EGCG also demonstrated efficiency against melanoma cells [138]. After treatment with EGCG-encapsulated chitosan NPs, a higher cytotoxic effect against Mel 928 human melanoma cells was observed with regulation of intrinsic apoptotic pathways and induction of cell cycle arrest with a dose advantage over free EGCG. These results were supported by the *in vivo* tests performed in a melanoma xenograft model where it was shown that oral administration of encapsulated EGCG was able to inhibit tumor growth and induce the intrinsic apoptotic pathway and cell cycle arrest.

Hu *et al.* [139] reported the use of genipicin-crosslinked caseinophosphopeptide (CPP)–chitosan NPs for encapsulation of EGCG. Cross-linking of the NPs with genipicin increased the stability of the nanocarriers at different pH values, and at simulated gastric and intestinal fluid (SGF and SIF). Alterations in the crosslinking degree of the NP enabled the modulation of the release profile of EGCG. This release rate was found to be higher in the SIF than in the SGF, which is appropriate for an oral delivery system. *In vitro* test with gastrointestinal cancer cell line BGC823 demonstrated that encapsulated EGCG retained its anti-tumoral activity.

Shutava *et al.* [8] synthesized gelatin-based NPs with or without a coating of polyelectrolytes polystyrene sulfonate/polyallylamine hydrochloride produced through the layer-by-layer technique. Gelatin NPs revealed a more sustained release of EGCG as compared with uncoated NPs. Encapsulated EGCG maintained its biological activity, being able to inhibit hepatocyte growth factor (HGF) and subsequent activation of cell signaling pathways responsible for cell invasion in breast cancer cell line MDA-MD-23.

### 3.3. Liposomes

Liposomes are vesicles forming a membrane-like phospholipid bilayer enclosing an aqueous compartment [140]. These structural properties enable the encapsulation of both lipophilic and hydrophilic drugs [141]. In addition, liposomes are biodegradable and present minimal levels of toxicity [140]. Few studies have used these nanocarriers for delivery of EGCG to cancer cells. Results are summarized in Table 3.

Fang *et al.* [142] developed liposomal formulations with EGCG and other catechins for topical and intra-tumoral administration to treat BCC (basal cell carcinoma) in female nude mice. The authors concluded that intra-tumoral injection of liposomes was the most effective route to reach cancer cells, promoting a great amount of EGCG deposition in tumor tissues. The same group reported the use of liposomal formulations for BCCs treatment *in vivo* after intra-tumoral administration [140]. Nanoencapsulation significantly increased EGCG stability compared to free drug, which, according to the authors, may indicate that liposomes protect EGCG from oxidation and degradation. The synthesized liposomes also enabled higher EGCG accumulation in tumor tissues and induced higher levels of BCC cell death compared to the non-encapsulated EGCG treatment at lower concentrations [140].

In work published by de Pace *et al.* [23], EGCG was encapsulated in the hydrophilic core of nanoliposomes formed by cholesterol and phosphatidylcholine and coated with 0.2% of chitosan. *In vitro* results demonstrated that these NPs significantly enhanced EGCG stability and prevented its premature degradation in both PBS and cell culture mediums, when compared to free EGCG which was degraded much faster. In addition, nanoencapsulation promoted a more extended release and a higher EGCG content in MCF7 breast cancer cells compared to free EGCG. Differences in EGCG cellular content were also detected after treatment with both chitosan-coated and non-coated nanoliposomes suggesting that chitosan increases cell absorption. A dose of 10 mM of chitosan-coated liposomes also revealed significant anti-proliferative and pro-apoptotic effects with a decrease of 40% of MCF7 cells’ proliferation compared with native EGCG and induction of 27% of MCF7 cell apoptosis.

More recently, Ramadass *et al.* [141] developed a liposomal co-delivery system comprising EGCG and paclitaxel for invasive cancer therapy using MDA-MB-231 breast cancer cell line. The results proved that this synergistic combination was effective in inducing cancer cell apoptosis and inhibiting cell invasion, which was demonstrated by an increase in caspase-3 activity and a decrease in MMP expression. These effects were higher in comparison with both paclitaxel and EGCG individual effects.

### 3.4. Other Type of NPs

A large variety of other different materials can be used for the design of nanoparticles. Encapsulation of EGCG for the purpose of cancer therapy using different materials, including carbohydrates, transition metals, inorganic materials and lipids are summarized in Table 4.

Rocha *et al.* [143] reported the encapsulation of EGCG into carbohydrate NPs composed of gum arabic and maltodextrin, whose properties enables them to protect the drug from oxidation. This nanocarrier promoted a reduction in cell viability in Du145 human prostate cancer cells and an induction of caspase-3 activation, and hence, apoptosis. These effects were higher comparing to free EGCG at low concentrations.

Zhou *et al.* [144] developed an anti-liver cancer therapy based on ruthenium NPs loaded with luminescent ruthenium complexes using EGCG as reducing and capping agent. Functionalization with EGCG was performed due to its high affinity to 67LR overexpressed in Hepatocellular carcinoma cells (HCC). *In vitro* results showed that the synthesized NPs had high specificity to liver cancer cells (SMMC-7721 HCCs) and their route of internalization was endocytosis mediated by 67LR. These NPs induced high levels of cytotoxicity, cell migration inhibition and induction of oxidative stress in HCC, while no harmful effects were detected in normal L-02 cells. The *in vivo* assay performed in a liver tumor xenograft model showed that intra-tumoral injection of EGCG functionalized nanocarriers could significantly inhibit tumor growth.

In a recent study developed by Shafiei *et al.* [5] EGCG was incorporated in Ca/Al-NO3 Layered double hydroxide (LDH) NPs using co-precipitation and ion-exchange techniques. The *in vitro* results revealed a higher anti-tumoral activity of the EGCG-LDH nanohybrid in a prostate cancer cell line (PC3), with a five-fold dose advantage over native ECGC and a longer release period compared to physical mixture of LDH and EGCG.

In these studies different types of nanoparticles were used including gold, polymeric, liposomes, metallic and carbohydrate-based. The majority of the studies have focused on polymeric NPs and liposomes, possibly due to their beneficial properties such as biocompatibility. A wide range of sizes was found varying from 20 to 1200 nm, although the majority of NPs were smaller than 250 nm. Different zeta potentials were also found, varying from positive (+30) to negative (−41). Encapsulation efficiencies were in general high, above 60%. EGCG anticancer activity was tested mainly in breast and prostate cancer models. Overall, the different types of nanoparticles promoted an enhancement of EGCG’s bioavailability, stability and release profile as well as improvement of its anticancer activity compared to free catechin. In some studies, surface functionalization increased some of these characteristics particularly the release profile and the bioavailability. Moreover, the use of targeting ligands described on some of the works, contributed to increase EGCG specificity and anti-tumoral activity. Two of the studies have addressed an interesting topic, which is the combination of EGCG with common cytostatic drugs, demonstrating their synergistic effect, which is encouraging for future chemotherapy approaches. Most studies, however, have not revealed whether the nanoparticles could protect EGCG from degradation and oxidation. This would be particularly relevant since this compound is very susceptible to oxidation, specially in alkaline environments [145,146]. Future studies should address this issue and evaluate the capacity that different nanoparticles have to protect EGCG from oxidation and premature degradation.

## 4. Conclusions

EGCG is the major bioactive component in green tea with many health benefits, including anti-cancer activity, which has been demonstrated in *in vitro* and *in vivo* models and corroborated by some clinical and epidemiological studies. Despite that, this catechin is still not currently used in clinical settings due to its limited bioavailability and stability. In order to overcome these limitations, several studies have been developed applying the concept of nanochemoprevention, the use of nanotechnology to improve the pharmacokinetic and pharmacodynamic of chemopreventive agents to manage cancer. In these studies, different types of nanoparticles including gold, polymeric, metallic, carbohydrate-based and liposomes were used as delivery vehicles of EGCG. In the majority of these studies, the size of the nanoparticles was below 250 nm and encapsulation efficiencies were higher than 60%. The results revealed that EGCG nanoparticles promoted prolonged circulation time in blood, increased cell internalization in tumor sites and inhibited tumor growth both *in vitro* and *in vivo* predominantly in breast and prostate cancer models. Surface functionalization was employed to enhance drug release, cell uptake and intestinal absorption. The use of targeting ligands further increased cancer cell specificity and improved the anti-tumor effects of EGCG. Some studies reported the combination therapy of EGCG with cytostatic agents, emphasizing the synergistic effect of the two compounds. These advances in EGCG nanodelivery systems highlight the importance of nanotechnology in the enhancement of EGCG anti-cancer activities and hold great promise for upcoming clinical applications. With this approach, it is expected that, in the future, EGCG could be commercially produced by nutraceutical and dietary supplement industries as innovative supplements for cancer prevention. In addition, when combined with conventional cytostatic drugs, EGCG may provide a useful contribution to cancer treatments. This synergistic association is expected to increase the effectiveness of the drug and decrease the administered doses, hence, minimizing its adverse side effects, which will greatly improve the efficiency of future cancer therapies and the quality of life of cancer patients.

## Figures and Tables

**Table 1:** Gold nanoparticles used as EGCG nanocarriers for cancer therapy.

Composition	Size (nm)	Zeta Potential (mV)	LC (%)	EE (%)	Route of Administration	*In Vitro*/*In Vivo* Results	Reference
Gold (EGCG/pNG 50 μM: 1.5 ppm)	20–1200	+21 ± 5	N/A	N/A	Oral Intra-tumoral or intra-peritoneal	High cytotoxicity towards bladder cancer cells (MBT-2) Marked reduction in tumor volume in bladder cancer xenograft model further accentuated via the intra-tumoral and intra-peritoneal administration route	[130]
Gold (EGCG/pNG 50 μM: 2.5 ppm)	64.7	−3.36	27	N/A	intra-tumoral	High cytotoxicity towards B16F10 murine melanoma cells Reduction in tumor volume in a mouse melanoma model	[125]
Gold	25.55 ± 7.26	N/A	N/A	N/A	N/A	Retention of EGCG’s anti-oxidant activity Induction of apoptosis in neuroblastoma SH-SY5Y-CFP-DEVD-YFP cells	[131]
Gold	45	+43	N/A	N/A	N/A	High toxicity towards EAC cells and protection of normal mouse hepatocytes	[11]

**Table 2:** Polymeric nanoparticles used as EGCG nanocarriers for cancer therapy.

Composition	Size (nm)	Zeta Potential (mV)	LC (%)	EE (%)	Route of Administration	*In Vitro*/*In Vivo* Results	Reference
PLGA-PEG	80.53 ± 15	N/A	N/A	9.61 ± 0.7	N/A	Increased cytotoxicity towards PSMA-positive LNCaP prostate cancer cell line	[135]
PLGA	127.2 ± 12	−24.5 ± 1.89	N/A	6	N/A	Increase in DNA damage levels of oxaliplatin- and satraplatin-treated lymphocytes from colorectal and healthy cancer patients	[132]
PLGA-casein	190–250	−41 ± 3.4	N/A	76.8 ± 9.1	N/A	Inhibition of NF-κB signaling Enhanced cytotoxicity towards breast cancer cells (MDA-MB-231 cell line and patient-derived cells)	[136,137]
PLA-PEG	260	−7.92	N/A	N/A	Intra-tumoral	High induction of apoptosis in prostate cancer PC3 cell line; inhibition of angiogenesis Significant decrease in tumor size in prostate cancer xenograft model	[25]
Chitosan	150–200	N/A	N/A	10	Oral	Higher inhibiton of tumor growth in prostate cancer xenograft model Inhibition of cancer cell proliferation and angiogenesis.	[126]
Chitosan	N/A	N/A	N/A	N/A	Oral	High cytoxicity against Mel 928 human melanoma cells Inhibition of tumor growth in melanoma xenograft model	[138]
CPP-chitosan	245.3 ± 18.3	32.4 ± 6.1	N/A	71	N/A	Higher stability in simulated GI tract conditions Maintenance of EGCG anti-tumoral activity against gastrointestinal cancer cell line BGC823	[139]
Gelatin	200	N/A	N/A	20–70	N/A	Sustained release of EGCG Ability to inhibit HGF in MDA-MD-231 breast cancer cell line	[8]

**Table 3:** Liposomes used as EGCG nanocarriers for cancer therapy.

Composition	Size (nm)	Zeta Potential (mV)	LC (%)	EE (%)	Route of Administration	*In Vitro*/*In Vivo* Results	Reference
Liposomes	157.4 ± 2.9	−7.2 ± 0.7	N/A	36.3 ± 5.7	Topic and intra-tumoral	Great amount of EGCG deposition in tumor tissues in BCC model in female nude mice	[142]
	268.9 ± 16.7	−66 ± 2.2		89.7 ± 0.4			
Liposomes	104.6–378.2	−0.9 ± 0,4	N/A	99.6 ± 0.1	Intra-tumoral	Higher EGCG accumulation in BCCs cells and higher apoptosis induction compared to free EGCG	[140]
		−36.1 ± 1.7		84.6 ± 3.8			
Chitosan-coated liposomes	85 ± 6.6	16.4 ± 2.8	3	90	N/A	High anti-proliferative and pro-apoptotic effects in MCF7 breast cancer cell line	[23]
Liposomes	126.7 ± 4.3	−37.5	N/A	60.21 ± 1.59	N/A	MDA-MB-231 breast cancer cell apoptosis and cell invasion inhibition	[141]

**Table 4:** Nanoparticles designed with various materials used as EGCG nanocarriers for cancer therapy.

Composition	Size (nm)	Zeta Potential (mV)	LC (%)	EE (%)	Route of Administration	*In Vitro*/*In Vivo* Results	Reference
Maltodextrin-gum arabic	120 ± 28	−12.3 ± 0.8	N/A	85 ± 3	N/A	Higher reduction in cell viability in Du145 human prostate cancer cells	[143]
Ruthenium	73.59	−17.9	N/A	N/A	Intra-tumoral	Induction of cancer cell apoptosis, oxidative stress and inhibition of migration Tumor growth inhibition in liver cancer xenograft model	[144]
Ca/Al-NO3 LDH	N/A	+30.6	N/A	N/A	N/A	Enhanced anti-tumoral activity of EGCG in PC3 prostate cancer cell line	[5]
